# IL-17C-mediated innate inflammation decreases the response to PD-1 blockade in a model of Kras-driven lung cancer

**DOI:** 10.1038/s41598-019-46759-8

**Published:** 2019-07-17

**Authors:** Felix Ritzmann, Christopher Jungnickel, Giovanna Vella, Andreas Kamyschnikow, Christian Herr, Dong Li, Michael M. Menger, Adrian Angenendt, Markus Hoth, Annette Lis, Robert Bals, C. Beisswenger

**Affiliations:** 1 Department of Internal Medicine V – Pulmonology, Allergology and Respiratory Critical Care Medicine, Saarland University, 66421 Homburg, Germany; 2 Department of Clinical Laboratory, Shanghai Tongji Hospital, Tongji University School of Medicine, 200065 Shanghai, China; 3 Institute for Clinical and Experimental Surgery, Saarland University Medical Center, 66421 Homburg, Germany; 4 Biophysics, Center for Integrative Physiology and Molecular Medicine, School of Medicine, Saarland University, Homburg, 66421 Germany

**Keywords:** Non-small-cell lung cancer, Cancer microenvironment

## Abstract

Chronic obstructive pulmonary disease (COPD) is associated with neutrophilic lung inflammation and CD8 T cell exhaustion and is an important risk factor for the development of non-small cell lung cancer (NSCLC). The clinical response to programmed cell death-1 (PD-1) blockade in NSCLC patients is variable and likely affected by a coexisting COPD. The pro-inflammatory cytokine interleukin-17C (IL-17C) promotes lung inflammation and is present in human lung tumors. Here, we used a Kras-driven lung cancer model to examine the function of IL-17C in inflammation-promoted tumor growth. Genetic ablation of *Il*-*17c* resulted in a decreased recruitment of inflammatory cells into the tumor microenvironment, a decreased expression of tumor-promoting cytokines (e.g. interleukin-6 (IL-6)), and a reduced tumor proliferation in the presence of *Haemophilus influenzae*- (NTHi) induced COPD-like lung inflammation. Chronic COPD-like inflammation was associated with the expression of PD-1 in CD8 lymphocytes and the membrane expression of the programmed death ligand (PD-L1) independent of IL-17C. Tumor growth was decreased in *Il*-*17c* deficient mice but not in wildtype mice after anti-PD-1 treatment. Our results suggest that strategies targeting innate immune mechanisms, such as blocking of IL-17C, may improve the response to anti-PD-1 treatment in lung cancer patients.

## Introduction

Lung cancer is a deadly disease responsible for more than one million deaths per year worldwide. COPD is an important risk factor for lung cancer and up to 70% of lung cancer patients have a coexisting COPD^1–3^. COPD is characterized by persistent inflammation of the lung which is further increased during exacerbations^4,5^. Stable CODP patients are frequently colonized with Gram-negative bacteria (e.g. NTHi), which contributes to neutrophilic lung inflammation, tissue destruction, and loss of lung function^5,6^. A recent study showed that neutrophils also dominate the immune cell composition in NSCLC^7^.

There is a causal relation between inflammation and the development of cancer^8^. In preclinical studies, it has been shown that in myeloid cells the nuclear factor-κB (NF-κB) pathway promotes cigarette smoke-induced release of tumor-promoting cytokines, such as TNF-α and IL-6, and lung tumorigenesis^9–11^. Preclinical studies also have shown that COPD-like airway inflammation induced by aerosolized NTHi strongly increases tumor growth in *Kras* mouse models through the activation of Toll-like receptor (TLR) signaling, the recruitment of neutrophils into the tumor microenvironment, and inflammatory mediators (e.g. IL-17A, IL-6)^12–17^. In addition, we have shown that acute lung inflammation promotes metastatic tumor growth and the recruitment of neutrophils into the tumor microenvironment through TLR signaling and the pro-inflammatory cytokine IL-17C^18,19^.

Immune checkpoint inhibitors that block the PD-1 pathway have shown encouraging results in NSCLC patients. However, the outcome for patients suffering from NSCLC still remains poor and many patients do not benefit from PD-1 therapy^20–22^. There is evidence that chronic inflammation of the lung results in the activation of the PD-1 pathway in NSCLC patients. COPD is associated with increased CD8 T cell exhaustion and an increased sensitivity of NSCLC patients to immune checkpoint inhibitors^20,23,24^. Clinical observations indicate an improved response to anti-PD-1 or anti-PD-L1 therapy in NSCLC patients with a coexisting COPD^20,23^. Therefore, COPD-linked inflammation likely affects the clinical response to PD-1 blockade and NSCLC patients with a coexisting COPD may qualify for the treatment with immune checkpoint inhibitor^20,23,24^.

The pro-inflammatory cytokine IL-17C is expressed by non-hematopoietic cells, such as epithelial cells, regulates innate immune functions, and is present in human lung tumors and in bronchial biopsies from COPD patients^18,25–29^. Moreover, cultured airway epithelial cells obtained from COPD patients secret increased amounts of IL-17C compared to cells from non-COPD patients^30^. We therefore examined the function of IL-17C in inflammation-promoted tumor growth in a Kras-driven lung cancer model. We provide evidence that ablation of IL-17C decreases inflammation-induced tumor growth and enhances the response to anti-PD-1 antibody treatment.

## Results

### IL-17C promotes inflammation-induced tumor proliferation

**Figure 1:**
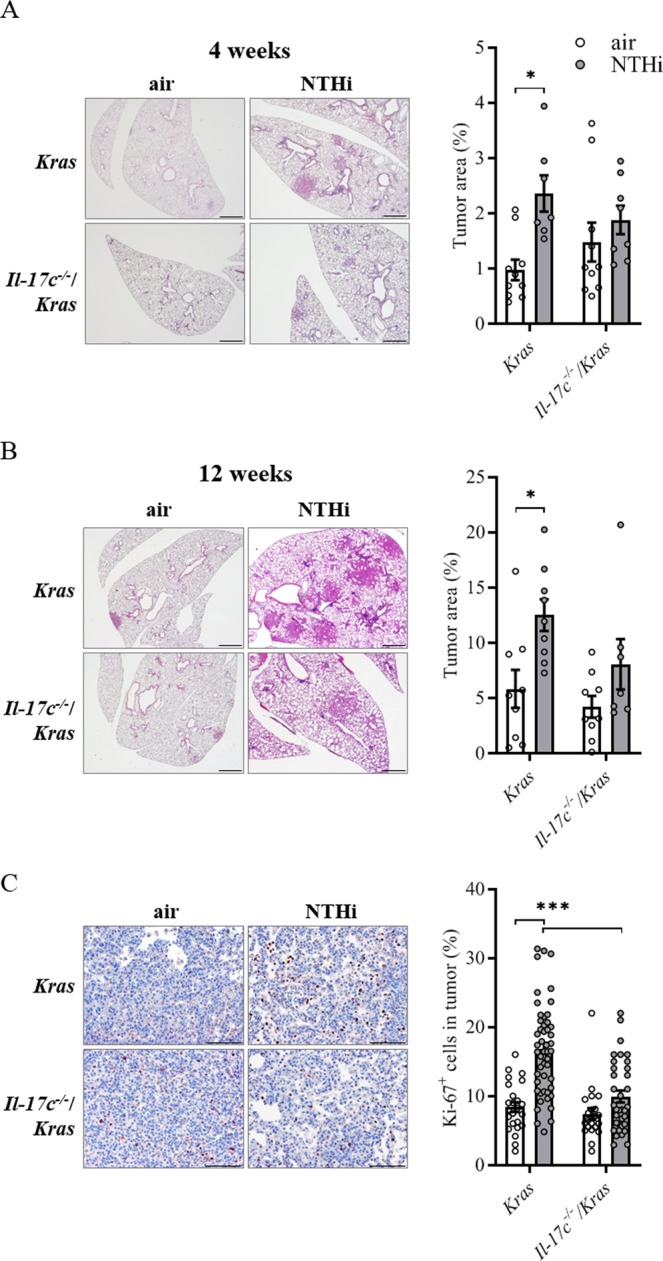
Ablation of *Il*-*17c* decreases NTHi-induced tumor progression. (**A**) Representative microscopic pathology and percentage of lung area covered by tumor lesions after exposure to NTHi for four weeks. (**B**) Representative microscopic pathology and percentage of lung area covered by tumor lesions after exposure to NTHi for twelve weeks. Scale bars: 1000 µm. (**C**) Representative IHC analysis of Ki-67 and Ki-67 index after exposure to NTHi for four weeks. Scale bars: 100 µm. Data were compared by Two-way ANOVA with Bonferroni post-test and are shown as the mean ± SEM. *p < 0.05, ***p < 0.001.

NTHi is associated with lung inflammation in stable COPD patients and during exacerbations^5^. To study the function of IL-17C in the progression of Kras-induced cancer lesions in a model of COPD-like inflammation^15,16^, we crossed *Kras*^*G12D*^ mice with *Il*-*17c*-deficient mice to obtain *Kras* mice deficient for IL-17C (*Il*-*17c*^−/−^/*Kras* mice) and exposed mice to NTHi three times a week for four or twelve weeks. Microscopic analysis showed that exposure to NTHi for 4 and 12 weeks resulted in a significantly increased lung area covered by tumor lesions (Fig. 1A,B) and in a significantly increased average size (Fig. S1) of the tumor lesions in *Kras* mice. There was no significant difference in tumor burden between NTHi-exposed *Il*-*17c*^−/−^/*Kras* and *Il*-*17c*^−/−^/*Kras* control mice. However, the difference in the area covered by tumor lesions and average size did not reach statistical significance between NTHi-exposed *Kras* and *Il*-*17c*^−/−^/*Kras* mice. Ki-67 staining showed that NTHi-induced inflammation of the lung resulted in an increased tumor cell proliferation in an IL-17C-dependent manner. The fraction of Ki67-positive tumor cells among 100 tumor cells was significantly increased in tumors of *Kras* mice compared with *Il*-*17c*^−/−^/*Kras* mice after four weeks of NTHi exposure (Fig. 1C).

**Figure 2:**
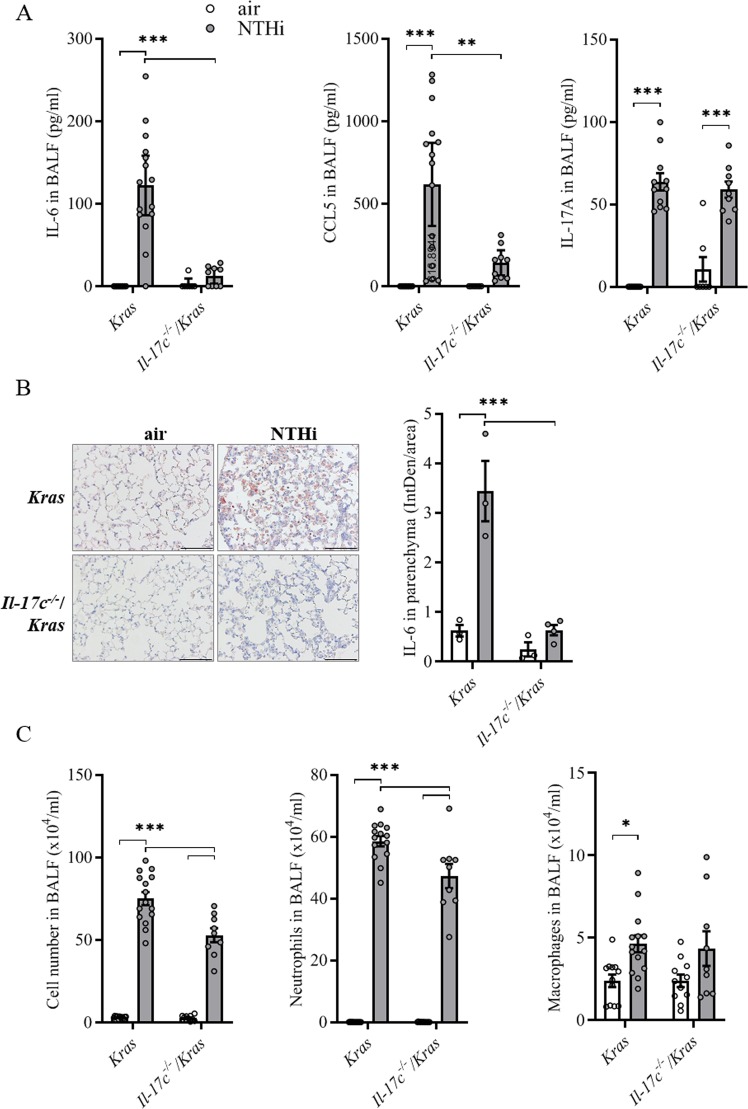
IL-17C contributes to chronic inflammation of the lung. *Kras* mice and *Il*-*17c*^−/−^/*Kras* mice were exposed to NTHi for 4 weeks. (**A**) Concentrations of IL-6, CCL5, and IL-17A in BAL fluids. (**B**) Representative IHC of IL-6 and quantification of the IL-6 relative intensity in parenchyma. Scale bars: 100 µm. (**C**) Numbers of total inflammatory cells, neutrophils, and macrophages were determined in BAL fluids. Data were compared by Two-way ANOVA with Bonferroni post-test and are shown as the mean ± SEM. *p < 0.05, **p < 0.01, and ***p < 0.001.

We further analyzed the pulmonary inflammation after exposure to NTHi for 4 weeks. Previous studies showed that the pleiotropic pro-inflammatory cytokine IL-6 promotes lung cancer growth in Kras-driven mouse models and that the expression of IL-6 is increased in human lung tumors^17,31^. We found that exposure to NTHi for 4 weeks resulted in significantly increased concentrations of IL-6 and CCL5 in lungs of *Kras* mice in an IL-17C-dependent manner (Fig. 2A). The concentrations of IL-6 and CCL5 were significantly increased in BAL fluids obtained from *Kras* mice compared with *Il*-*17c*^−/−^/*Kras* mice. However, there was no difference in the concentrations of IL-17A in BAL fluids between Kras and *Il*-*17c*^−/−^/*Kras* mice. In addition, IHC analysis showed that the expression of IL-6 was significantly increased in lungs of *Kras* mice compared with *Il*-*17c*^−/−^/*Kras* mice after four weeks of NTHi exposure (Fig. 2B). Numbers of total inflammatory cells and neutrophils were reduced in BAL fluids of *Il*-*17c*^−/−^/*Kras* mice after four weeks of NTHi exposure, whereas numbers of macrophages were not affected by the deficiency for IL-17C (Fig. 2C).

### IL-17C promotes the rapid recruitment of neutrophils into the tumor microenvironment

**Figure 3:**
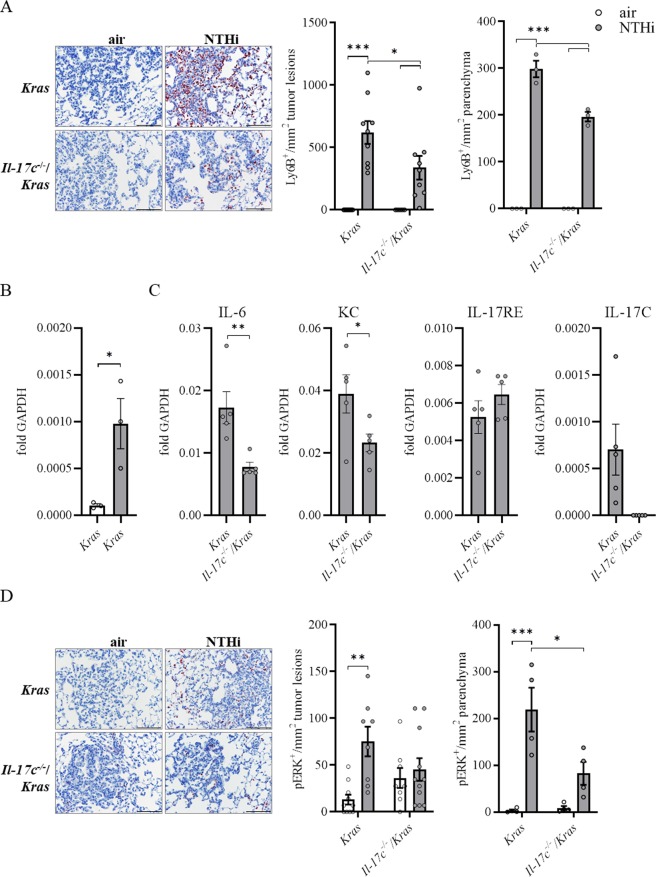
*Il*-*17c* deletion decreases rapid recruitment of inflammatory cells into the tumor microenvironment and ERK activation. (**A**) Representative IHC of Ly6B and quantification of the Ly6B positive cells in tumor lesions and parenchyma four hours after the exposure to a single dose of NTHi. Data were compared by Two-way ANOVA with Bonferroni post-test and are shown as the mean ± SEM. (**B**) Relative expression of IL-17C in lung tissue four hours after the exposure to NTHi. Data were compared by unpaired Student’s t-test and are shown as the mean ± SEM. (**C**) Relative mRNA expression of IL-6, KC, IL-17RE, and IL-17C in lung tissue 24 hours after the exposure to NTHi. Data were compared by unpaired Student’s t-test. (**D**) Representative IHC of phosphorylated Erk (p-Erk) and quantification of p-Erk positive cells in tumor lesions and parenchyma four hours after the exposure to NTHi. Data were compared by Two-way ANOVA with Bonferroni post-test and are shown as the mean ± SEM. *p < 0.05, **p < 0.01, and ***p < 0.001. Scale bars: 100 µm.

Recent studies showed that myeloid cells such as neutrophils strongly infiltrate lung tumors and that tumor-associated inflammation is a key driver of cancer growth^7,8^. We have shown before that NTHi-induced neutrophilic inflammation accelerates the proliferation of Kras-driven tumors in a TLR-dependent manner and that IL-17C promotes the recruitment of neutrophils into Lewis lung carcinoma cell tumors^16,18^. We therefore examined whether IL-17C mediates the recruitment of neutrophils into the microenvironment of Kras-driven tumors. We analyzed the expression of Ly6B, a marker for neutrophils and inflammatory monocytes^32^, in tumor lesions and in the parenchyma by immunohistochemistry (IHC) four hours after a single exposure to NTHi. There were nearly no Ly6B^+^ cells present in the tumor lesions and parenchyma of *Kras* and *Il*-*17c*^−/−^/*Kras* control mice, whereas NTHi-induced inflammation resulted in the recruitment of segmented Ly6B^+^ cells into the tumor microenvironment (Fig. 3A). Remarkably, the density of Ly6B^+^ cells was twice as high in the tumor lesions compared to the parenchyma and significantly decreased in *Il*-*17c*^−/−^/*Kras* mice.

We also determined the expression of *Il*-*17c* at the transcriptional level. The expression of IL-17C was increased in lungs of *Kras* mice four hours after exposure to NTHi (Fig. 3B). In addition, the relative mRNA expression of the cytokines IL-6 and keratinocyte-derived chemokine (KC) was significantly decreased in lungs of *Il*-*17c*^−/−^/*Kras* mice 24 hours after the exposure to NTHi whereas the expression of the IL-17C receptor IL-17RE was not affected by the deficiency for IL-17C (Fig. 3C). IL-17C was not detectable in *Il*-*17c*^−/−^/*Kras* mice, verifying the expected result

It has been shown that IL-17 cytokines signal synergistically with other stimuli through the activation of mitogen-activated protein kinase (MAPK) pathways^33^. To examine whether IL-17C mediates the activation of the MAP kinase ERK we determined ERK phosphorylation four hours after the exposure to NTHi. IHC analysis showed that the NTHi-induced inflammation resulted in increased numbers of p-ERK-positive cells in the tumor microenvironment of *Kras* mice, which was not seen in *Il*-*17c*^−/−^/*Kras* mice (Fig. 3D). To test whether IL-17C directly activates ERK-signaling in epithelial-derived cancer cell lines and in primary epithelial cells, we stimulated murine (LA4) and human (Calu-3) cancer cell lines as well as primary human bronchial epithelial cells (HBEC) with IL-17C, NTHi, and the combination of IL-17C and NTHi. Western blot analysis revealed increased levels of p-ERK in lysates of the cancer cell lines after 30 and 60 minutes of incubation with IL-17C and the combination of NTHi (Fig. S2).

Taken together, our data suggest that IL-17C promotes proliferation of tumor cells through the regulation of innate immune mechanisms in the tumor microenvironment.

### COPD-like inflammation results in the expression of PD-1 and PD-L1 independent of IL-17C

**Figure 4:**
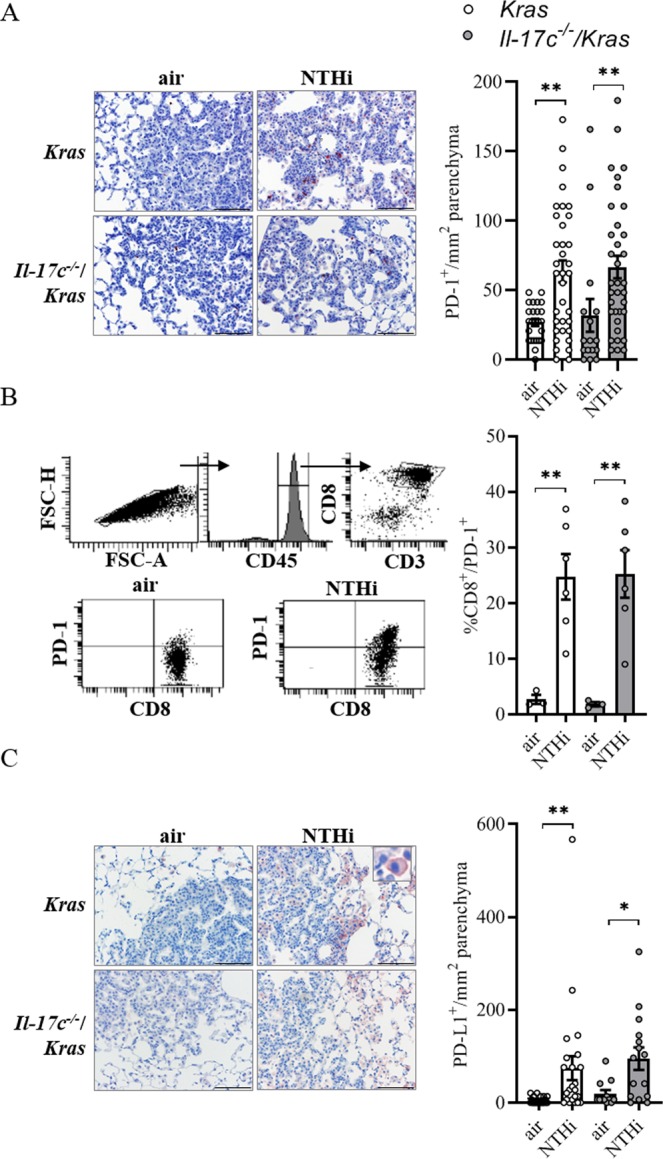
The expression of PD-1 is increased in lymphocytes after chronic exposure to NTHi. *Kras* mice and *Il*-*17c*^−/−^/*Kras* mice were exposed to NTHi for 4 weeks. (**A**) Representative IHC of PD-1 and quantification of the PD-1 positive cells in cancerous lesions. (**B**) Gating strategy and relative abundance of PD-1 expressing CD8 T cells in the total lung. (**C**) Representative IHC of PD-L1 and quantification of the PD-L1 positive cells in cancerous lesions. Scale bars: 100 µm. Data were compared by unpaired Student’s t-test and are shown as the mean ± SEM. *p < 0.05 and **p < 0.01.

Recent studies indicate that the expression of PD-1 is increased in tumor-associated CD8 T cells of patients with COPD and that the sensitivity to PD-1 blockade is increased in lung cancer patients with COPD^20,23,24^. Moreover, myeloid cells such as myeloid-derived suppressor cells express PD-L1 in the tumor microenvironment of NSCLC^34^. Therefore, we sought to examine whether the PD-1 immune checkpoint is activated in our model of COPD-like inflammation. IHC staining showed that the numbers of tumor associated PD-1^+^ cells (Fig. 4A) as well as CD4^+^ and CD8^+^ cells (Fig. S3) were significantly increased after exposure to NTHi for four weeks in an IL-17C-independent manner. Flow cytometry analyses showed that exposure to NTHi for 4 weeks resulted in an increased proportion of PD-1 expressing CD8^+^ T cells in lungs of *Kras* and *Il*-*17c*^−/−^/*Kras* (Fig. 4B). Moreover, NTHi-induced inflammation resulted in a significantly increased membrane expression of PD-L1 in cells located at the edges of the tumor lesions (Fig. 4C). These data indicate that chronic inflammation as seen in COPD patients strongly activates the PD-1 immune checkpoint in the Kras-driven lung cancer model independent of IL-17C expression.

### Blockade of the PD-1 immune checkpoint results in reduced cancer growth in Il-17c deficient mice

**Figure 5:**
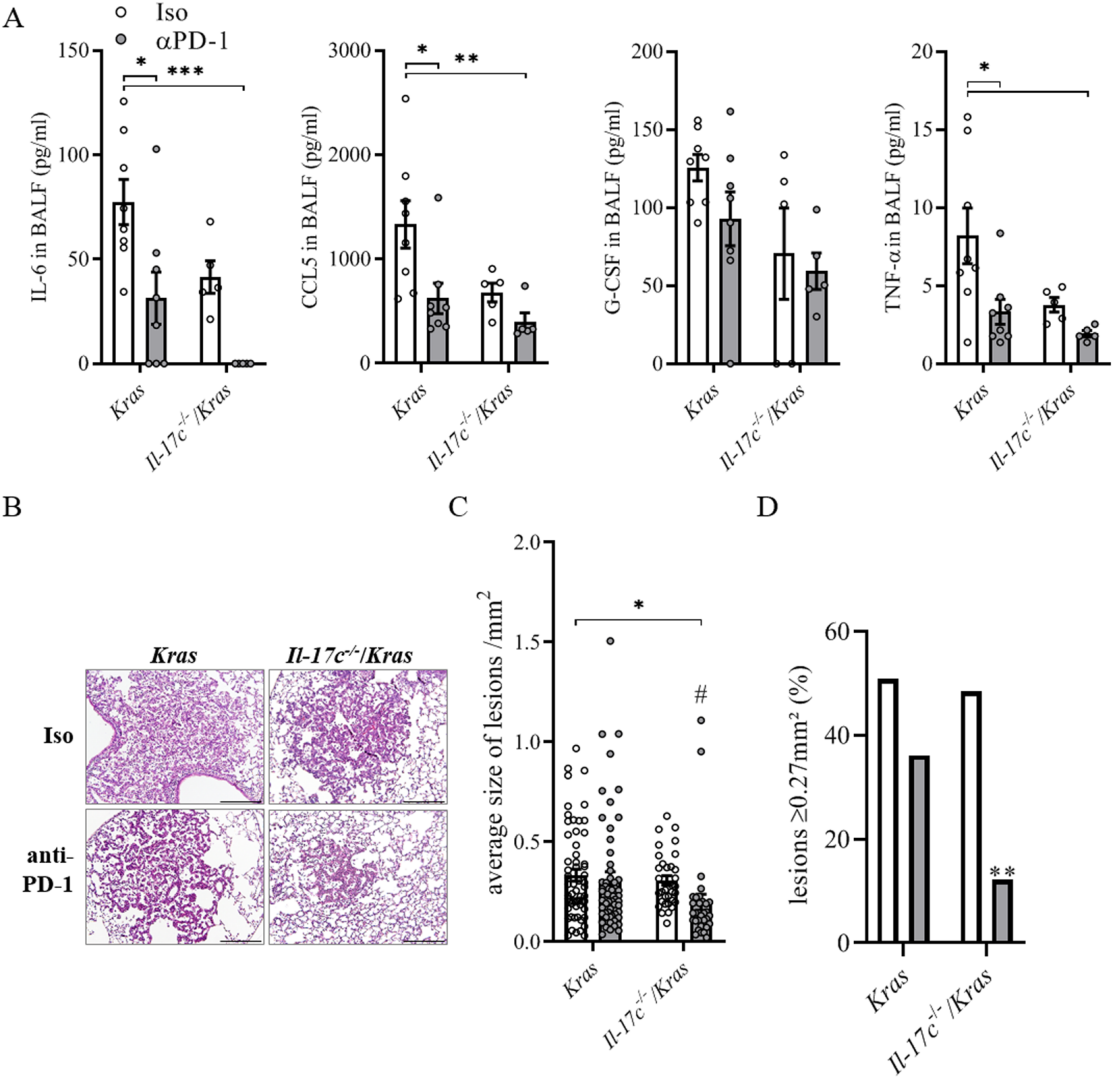
Anti-PD-1 treatment decreases lung inflammation and tumor growth in *Il*-*17c* deficient mice. *Kras* mice and *Il*-*17c*^−/−^/*Kras* mice were exposed to NTHi for 4 weeks and treated with an anti-PD-1 antibody or an isotope antibody during the exposure phase. (**A**) Concentrations of IL-6, CCL5, G-CSF, and TNF-α in BAL fluids. Data were compared by Two-way ANOVA with Bonferroni post-test and are shown as the mean ± SEM. *p < 0.05, **p < 0.01, and ***p < 0.001. Scale bars: 100 µm. (**B**) Representative microscopic pathology. Scale bars: 200 µm. (**C**) Average sizes of tumor lesions. Data were compared by Two-way ANOVA with Bonferroni post-test with *p < 0.05 or by Mann Whitney test on isotype-treated *Il*-*17c*^−/−^/*Kras* mice with ^#^p < 0.001. Data are shown as the mean ± SEM. (**D**) Percentage of tumor lesions larger than 0.27 mm^2^. Data were compared by Fisher’s exact test with **p < 0.01.

In Kras-driven lung cancer models, the tumor growth is hardly affected by PD-1 blockade without any further treatment such as radiotherapy^35,36^. We sought to examine whether blocking of PD-1 affects inflammation-induced tumor growth. Therefore, *Kras* and *Il*-*17c*^−/−^/*Kras* mice were exposed to NTHi for four weeks and treated with a PD-1-blocking antibody or an isotype antibody three times a week. The blockade of PD-1 resulted in decreased concentrations of the cytokines IL-6, CCL5, G-CSF, and TNF-α (Fig. 5A) and decreased numbers of neutrophils (Fig. S4) in BAL fluids of *Kras* and *Il*-*17c*^−/−^/*Kras* mice. Concentration of IFN-γ, IL-10, and IL-12p70 were below the detection limit of 3.35, 2.41, and 9.92 pg/ml, respectively (data not shown). IHC staining showed that numbers of tumor-associated CD4^+^ cells were reduced in anti-PD-1 treated *Kras* and *Il*-*17c*^−/−^/*Kras* mice (Fig. S5). Microscopic analysis illustrates that blocking of PD-1 resulted in smaller tumor lesions in *Il*-*17c*^−/−^/*Kras* mice but not in Kras mice (Fig. 5B). The average size of tumor lesions was indeed significantly reduced in anti-PD-1 treated *Il*-*17c*^−/−^/*Kras* mice compared to anti-PD-1 treated *Kras* mice or to *Il*-*17c*^−/−^/*Kras* mice treated with an isotype antibody (Fig. 5C). In addition, the percentage of tumor lesions larger than 0.27 mm^2^ was decreased in anti-PD-1 treated *Il*-*17c*^−/−^/*Kras* mice compared to all other conditions (Fig. 5D). These data indicate that deletion of IL-17C improves the response to anti-PD-1 treatment in our model of neutrophilic lung inflammation.

## Discussion

COPD and NSCLC are connected diseases and marked by the activation of innate immunity and the presence of inflammatory cells (e.g. neutrophils) in the lung and tumor tissue^7,8,20,23,24^. Here, we demonstrate that the pro-inflammatory cytokine IL-17C promotes the rapid recruitment of neutrophils into the tumor microenvironment, the expression of tumor-promoting-cytokines, and tumor proliferation in the presence of COPD-like lung inflammation. We found that chronic COPD-like inflammation results in the expression of PD-1 and PD-L1 in immune cells in the tumor microenvironment independent of IL-17C. We also demonstrate that treatment with a PD-1-blocking antibody decreases inflammation in the tumor microenvironment and reduces tumor growth in the absence of IL-17C. Thus, our work provides further evidence that COPD-linked inflammation promotes tumor proliferation through innate immune mechanisms and the activation of the PD-1 immune checkpoint in the tumor microenvironment.

We and others showed that COPD-like airway inflammation strongly increases Kras-driven lung cancer growth through the activation of innate immunity by TLRs and the recruitment of neutrophils into the tumor microenvironment^13–16^. Studies showed that IL-17C regulates innate immunity at epithelial surfaces and the recruitment of neutrophils into inflamed tissues^18,25–29,37^. We detected IL-17C in human lung cancer samples and showed that IL-17C mediates the recruitment of neutrophils and lung tumor growth in a metastatic lung cancer model of acute lung inflammation^18^. We therefore explored whether IL-17C-mediated activation of innate immune responses affects Kras-driven tumor growth in a model of chronic COPD-like inflammation. In line with the metastatic lung cancer model^18^, genetic ablation of *Il*-*17c* in *Kras* mice resulted in a decreased rapid recruitment of neutrophils into tumor lesions. Remarkably, the numbers of neutrophils were twice as high in the tumor lesions compared to the parenchyma and significantly decreased in mice deficient for IL-17C. There were only few neutrophils present in the absence of COPD-like inflammation. Therefore, we propose a disease model, in which activation of innate immune mechanisms through IL-17C promotes the recruitment of myeloid cells, such as neutrophils, into the tumor microenvironment. Moreover, the expression of the tumor-promoting cytokines IL-6, which has been shown to be regulated by IL-17C in different disease models^29,38–40^, and CCL5 was decreased in lungs of IL-17C deficient *Kras* mice. Studies showed that IL-6 enhances the progression of Kras-driven lung carcinogenesis. Ablation or blocking of IL-6 signaling resulted in reduced tumor growth in absence or presence of COPD-like airway inflammation and in mice constitutively expressing IL-17A in the lung epithelium^17,31,41–43^. In addition, CCL5 and IL-6 promote *Kras*-dependent lung cancer cell proliferation and migration^44^. Thus, the decreased expression of tumor-promoting cytokines may be one factor responsible for the decreased tumor-proliferation in *Il*-*17c* deficient mice.

IL-17 cytokines signal synergistically with other stimuli, such as TLR ligands, through the activation of MAP pathways^33^. As demonstrated for colonic epithelial cells we show in this study that IL-17C induces the rapid phosphorylation of ERK in cultured cancer cell lines and HBECs. In addition, numbers of phosphorylated ERK positive cells were reduced in lungs of *Il*-*17c* deficient mice. This suggests that IL-17C mediates the expression of tumor-promoting cytokines through the activation of MAP kinase signaling pathways.

IL-17A has been shown to promote tumorigenesis in the absence and presence of NTHi-induced inflammation in a Kras-dependent lung cancer model^12^. In contrast to IL-17A, deletion of IL-17C did not affect Kras-induced intrinsic inflammation and tumor cell proliferation within the observation period of 12 weeks. This suggests that IL-17C has a specific role in the promotion of lung cancer only in the presence of exogenous lung inflammation or insult, such as bacterial colonization in COPD patients. This difference between IL-17A and IL-17C, which both need the IL-17 receptor IL-17RA for the activation of target cells, likely relates to the cellular source of these cytokines. Chang *et al*. found enriched levels of Th17 cells in tumor tissue in the absence of external inflammation^12^, whereas Il-17C is expressed by epithelial cells and not by immune cells. Moreover, in the absence of NTHi-induced inflammation the expression of IL-17C was rather low.

As mentioned above, in non-treated *Kras* mice, tumor-associated neutrophils and tumor-promoting cytokines (e.g. IL-6 in BAL fluids) were almost not detectable. In addition, numbers of PD-1-expressing CD8^+^ cells and PD-L1-expressing cells in the tumor microenvironment were low. In opposite to non-treated Kras mice, chronic COPD-like lung inflammation resulted in a tumor micromilieu similar to that in NSCLC patients in different aspects. A recent study showed that PD-1 expression on CD4^+^ cells associates with the tumor size and that neutrophils are the most abundant immune cell type in NSCLC^7^. Neutrophils are among possible immunotherapeutic targets in addition to immune checkpoint inhibitors^7,45^. Like in NSCLC patients^7,20,23,24^, chronic COPD-like inflammation associated with a heterogeneous immune cell population characterized by the presence-of neutrophils, the expression of PD-1 in CD8 T cells and the presence of PD-1^+^, CD4^+^, and CD8^+^ cells in the tumor microenvironment.

There is evidence that COPD is associated with CD8 T cell exhaustion and that the PD-1 pathway is activated in NSCLC patients with a coexisting COPD^20,23,24^. Therefore, it is suggested that COPD-linked inflammation increases the sensitivity of NSCLC patients to PD-1/PD-L1 treatment. Indeed, first clinical studies indicate that NSCLC patients with a coexisting COPD qualify for therapies that block the PD-1/PD-L1 pathway^20,23^. In our model of COPD-like inflammation, IL-17C deficiency enhanced the response to PD-1 therapy without affecting the expression of PD-1 in CD8 lymphocytes and the membrane expression of PD-L1 in myeloid cells. Therefore, IL-17C-mediated expression of tumor-promoting cytokines (e.g. IL-6) seems to decrease the sensitivity to PD-1 blockade^45–47^. This assumption is supported by recent preclinical studies. Targeted inhibition of IL-6, for instances, enhanced the efficacy of anti-PD-1 therapy in pancreatic cancer models and in melanoma-bearing mice^46,47^. In addition, a recent study showed that IL-17A which shares the IL-17 receptor IL-17RA with IL-17C^48^ mediates resistance to PD-1 blockade in Kras mice expressing a conditional IL-17A allele^41^. In this model, forced expression of IL-17A promoted lung cancer growth through IL-6 and tumor-associated neutrophils^41^. Recent studies also showed that neutrophils limit anti-tumor immune responses by suppressing T cell activity through different mechanisms, such as the production of reactive oxygen species or arginase^49,50^. In addition, even though concentrations of IL-10 were below the detection in BAL fluids 4 weeks after exposure to NTHi, it is possible that the deficiency for IL-17C results in a decreased expression of anti-inflammatory cytokines, such as IL-10, at earlier time points, leading to an increased response to the anti-PD-1 treatment. Together, our data suggest that COPD-like inflammation associates with a tumor microenvironment that is required for an efficient anti-PD-1 therapy, but, at the same time, also promotes the expression of innate cytokines, such as IL-17C, that counteract the response to immune checkpoint inhibitors. Additional studies are needed to determine whether targeted inhibition of innate cytokines, such as IL-17C or IL-6, with therapeutic antibodies enhance the response to PD-1 blockade in lung cancer.

Even though the pulmonary inflammation was decreased in anti-PD-1 treated Kras mice, histologic analysis did not show any difference in the tumor burden between isotype and anti-PD-1 treated *Kras* mice. This may be due the relatively short observation period of four weeks. Differences in the tumor burden could become visible at later time points.

There is evidence for a relatively low immunogenicity of the tumors in of Kras-driven lung cancer models^35,36,51,52^. We suggest that, in our model of Kras-driven lung cancer, COPD-like lung inflammation also promotes tumor cell proliferation through tumor-promoting cytokines and inflammatory cells, such as neutrophils. Therefore, disease models using more immunogenic cancer cells are required to verify the effect of innate immune mechanisms on the response to anti-PD-1 treatment in the context with neoantigen-specific T cell and to explore whether strategies that combine immune checkpoint inhibitors with agents targeting innate immunity are beneficial for specific cohorts of NSCLC patients, such as NSCLC patients with a coexisting COPD.

## Methods

### Lung cancer model

C57BL/6 *Kras*-mice (K-ras^LA1^, own breeding) were crossed into *Il*-*17c*-deficient C57BL/6 mice (own breeding) initially obtained from the Mutant Mouse Resource and Research Center (MMRRC, USA) to obtain IL-17C deficient Kras (*Il*-*17c*^−/−^/*Kras*) mice^29,53^. All animal studies were approved by the “Landesamt für Verbraucherschutz des Saarlandes, Germany” in agreement with the national guidelines for animal treatment. The mice with the same genotypes were randomly chosen for the experimental groups. 8 to 10 weeks old female Kras and *Il*-*17c*^−/−^/*Kras* mice were exposed to NTHi as described before^16,18^. Briefly, the mice were exposed to heat-inactivated, sonicated NTHi (clinical isolate, protein concentration adjusted to 2.5 mg/ml in PBS) for 40 minutes per day in a plexi glass box connected to a Pari MASTER® nebulizer (Pari GmbH, Starnberg, Germany).

### Antibody-treatment

PD-1 blocking antibody (clone 29F.1A12) and isotype control (clone 2A3) were purchased from BioXcell (West Lebanon, NH, USA). Antibodies were administrated by intraperitoneal injection (200 µg in PBS per dose) in *Kras* and Il-17c^−/−^/*Kras* mice three times a week for four weeks.

### Histopathology

All histologic analysis were performed as described before^16,18,19^. Briefly, the lungs were fixed under a constant hydrostatic pressure of 30 cm for 15 minutes in PBS-buffered 4% formalin and pre-embedded in 1% agarose. The lungs were cut into regular slices to obtain at least three different sectional planes and embedded in paraffin. Paraffin sections were stained with haematoxilin-eosin (H&E). The slides were scanned on an Olympus BX51 microscope (Olympus Corporation, Shinjuku, Japan) equipped with an eight-position slide loader (Ludl Electronic products, ltd, Hawthorne, USA). Lesions were marked by hand and the size (mm^2^) of each individual tumor lesion was measured using the software cellSens Dimension (Olympus Corporation) blinded to the investigator. The total lung area was determined (mm^2^, Visiopharm Integrator System Version 4.2.7.0, Visiopharm, Hoersholm, Denmark) and the percentage of tumor area from total lung area was calculated. Following primary antibodies used for immunohistochemistry as described before^16,18,19^: anti-Ki67 (ab15580, Abcam, Cambridge, UK), anti-Ly-6B.2 (Clone 7/4, Serotec), p44/42 MAPK (Erk1/2, Cell Signaling Technology, CST, Cambridge, UK), anti-PD-1 and anti-PD-L1 (R&D Systems, Minneapolis, MN, USA), anti CD8 and anti CD4 (Abcam). Corresponding HRP-conjugated secondary antibodies (Histofine Simple Stain, Nichirei Biosciences Inc. Japan) were used. For immunohistochemistry cells were permeabilized with Tween-20 (0.5% in TBS buffer). Relative intensity of IL-6 staining was quantified using ImageJ software (National Institutes of Health, Bethesda, MD, USA). Ki-67 index was defined as Ki67-positive cells per 100 tumor cells.

### RT-PCR

RNA isolated from blood-free lungs (Trizol Reagent, Life Technologies, Carlsbad, CA, USA) was reversely transcribed using a cDNA Synthesis Kit (ThermoScientific, Frankfurt, Germany). qRT-PCRs (SYBR Kit, Bioline, Luckenwalde, Germany) were performed and analyzed with the ΔΔCT method as described before^18,54,55^. Specificity of amplification was controlled by gel electrophoresis and melt curve analysis.

### Cell line experiments

Calu-3 cells were cultured in DMEM-F12 (Thermo Fisher, Waltham, MA, USA) supplemented with 10% fetal calf serum (FCS, Thermo Fisher), 100 U/ml penicillin, and 100 U/ml streptomycin (Thermo Fisher, USA). LA-4 cells were cultured in F12 Nutrient mixture (Thermo Fisher), supplemented with 15% FCS, 100 U/ml penicillin, and 100 U/ml streptomycin (Thermo Fisher). FCS was reduced to 1% 24 hours before treatment. HBECs were isolated from large airways resected during surgery and cultured in airway epithelial cell medium (PromoCell, Heidelberg, Germany). Only cancer-free tissue was used for cell isolation. The protocol was approved by the Institutional Review Board of the Landesärztekammer des Saarlandes (ethics committee). Informed consent was obtained from the patients and all methods were performed in accordance with the relevant guidelines and regulations. NTHi were cultured in brain-heart infusion broth (Roth, Germany) supplemented with 2% Difco Supplement B (BD Biosciences, Heidelberg, Germany). The bacteria were washed, resuspended with PBS, and heat inactivated at 70 °C for 45 min. NTHi and recombinant IL-17C (murine and human, R&D Systems) were diluted in culture media as indicated in the figure legend.

Cells were lysed on ice for 30 min in cold RIPA-lysis Buffer with added phosphatase / protease inhibitors (Pierce Phosphatase Inhibitor Tablets Thermo Fisher; Protease Inhibitors complete Tablets, Roche Diagnostics, Mannheim, Germany). Protein concentrations were determined with a Pierce BCA-Protein Assay Kit (Thermo Fisher). Proteins (10 µg) were separated on a 4–12% SDS polyacrylamide gel. Proteins were transferred to a polyvinylidene difluoride membrane and probed for phospho-p44/42 (pErk1/2, CST). Blots were stripped and probed for p44/42 MAPK (Erk1/2, CST). The membranes were analyzed by enhanced chemiluminescence (BioRad, Dreieich, Germany) using appropriate peroxidase-conjugated secondary antibodies (Agilent, DAKO, Santa Clara, CA, USA). Densitometry was performed using ImageJ Software.

### Bronchoalveolar lavage

Bronchoalveolar lavage (BAL) fluids were obtained from mice as described before^16,18,56^. Briefly, numbers of total inflammatory cells in the BAL fluids were counted by using a hemocytometer (Innovatis AG, Reutlingen, Germany). Leukocyte subpopulations were differentiated by DiffQuick Staining (Medion Diagnostics, Miami, FL, USA) on Cytospins. G-CSF, IL-6, RANTES, TNF-α, and IL-17A were measured in BAL fluids using by Luminex Bead Based Multiplex Assay (R&D) on a MAGPIX System.

### Flow cytometry

The lungs were perfused with cold PBS through the left ventricle until the lungs were cleared from blood before enzymatic and mechanical disruption into single cell solution (Lung dissociation Kit, Miltenyi Biotec, Bergisch Gladbach, Germany). Single cell solutions were pretreated with Fc-Block (anti-mouse CD16/CD32, clone 2.4G2, BD Biosciences) and stained with fluorophore-conjugated antibodies (CD45.2, PerCPCy5.5; CD3, BD V500; CD4, APC-H7; CD8, FITC; PD-1, Alexa Fluor 647; isotype) for 15 min at RT. Samples were run on a FACS Canto II (BD Biosciences) and analyzed using FACSDiva Software (BD Biosciences).

### Statistical analysis

Comparisons between two groups were analyzed by appropriate parametric (Two-way ANOVA with Bonferroni post-test, student t-test) or nonparametric (Mann-Whitney) tests using the software Prism (GraphPad Software, San Diego, CA). The results were considered statistically significant for P < 0.05.

## Supplementary information

SUPPLEMENTARY INFO

## Data Availability

The datasets generated and analyzed during the current study are available from the corresponding author on reasonable request.
