# Supplementary material for: IL-17C-mediated innate inflammation decreases the response to PD-1 blockade in a model of Kras-driven lung cancer
Source: Sci Rep. 2019 Jul 17;9:10353. doi: 10.1038/s41598-019-46759-8 (PMC6637115; doi:10.1038/s41598-019-46759-8)
Supplement: Supplementary file 1 — SUPPLEMENTARY INFO [file 41598_2019_46759_MOESM1_ESM.pdf]

SUPPLEMENTARY INFORMATION

IL-17C-mediated innate inflammation decreases the response to PD-1 blockade in a model of Kras-driven lung cancer

Felix Ritzmann[1], Christopher Jungnickel[1], Giovanna Vella[1], Andreas Kamyschnikow[1], Christian Herr[1], Dong Li[2], Michael M. Menger[3], Adrian Angenendt[4], Markus Hoth[4], Annette Lis[4], Robert Bals[1], and Beisswenger C[1*]

[1] Department of Internal Medicine V – Pulmonology, Allergology and Respiratory Critical Care Medicine, Saarland University, 66421 Homburg, Germany

[2] Department of Clinical Laboratory, Shanghai Tongji Hospital, Tongji University School of Medicine, 200065 Shanghai, China

[3] Institute for Clinical and Experimental Surgery, Saarland University Medical Center, 66421 Homburg, Germany

[4] Biophysics, Center for Integrative Physiology and Molecular Medicine, School of Medicine, Saarland University, Homburg, 66421, Germany

*Corresponding author:

PD Dr. rer. physiol. Christoph Beisswenger

Department of Internal Medicine V

Universität des Saarlandes

Kirrberger Str. 100, Building 61.4

66421 Homburg/Saar, GERMANY

Tel.: (+49) 06841-16 47915, Fax: (+49) 06841-16 47918

Correspondence to christoph.beisswenger@uks.eu

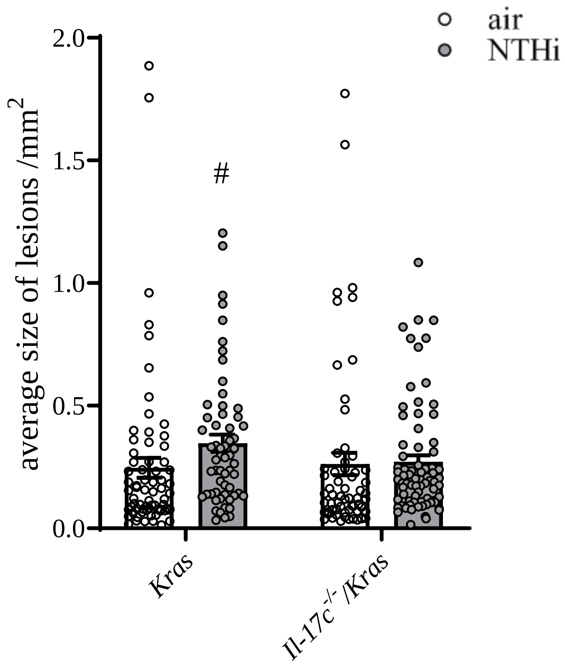

S1: Average size of tumor lesions after exposure to NTHi for four weeks. Data were compared by Mann Whitney test air-exposed Kras mice with #p < 0.001.

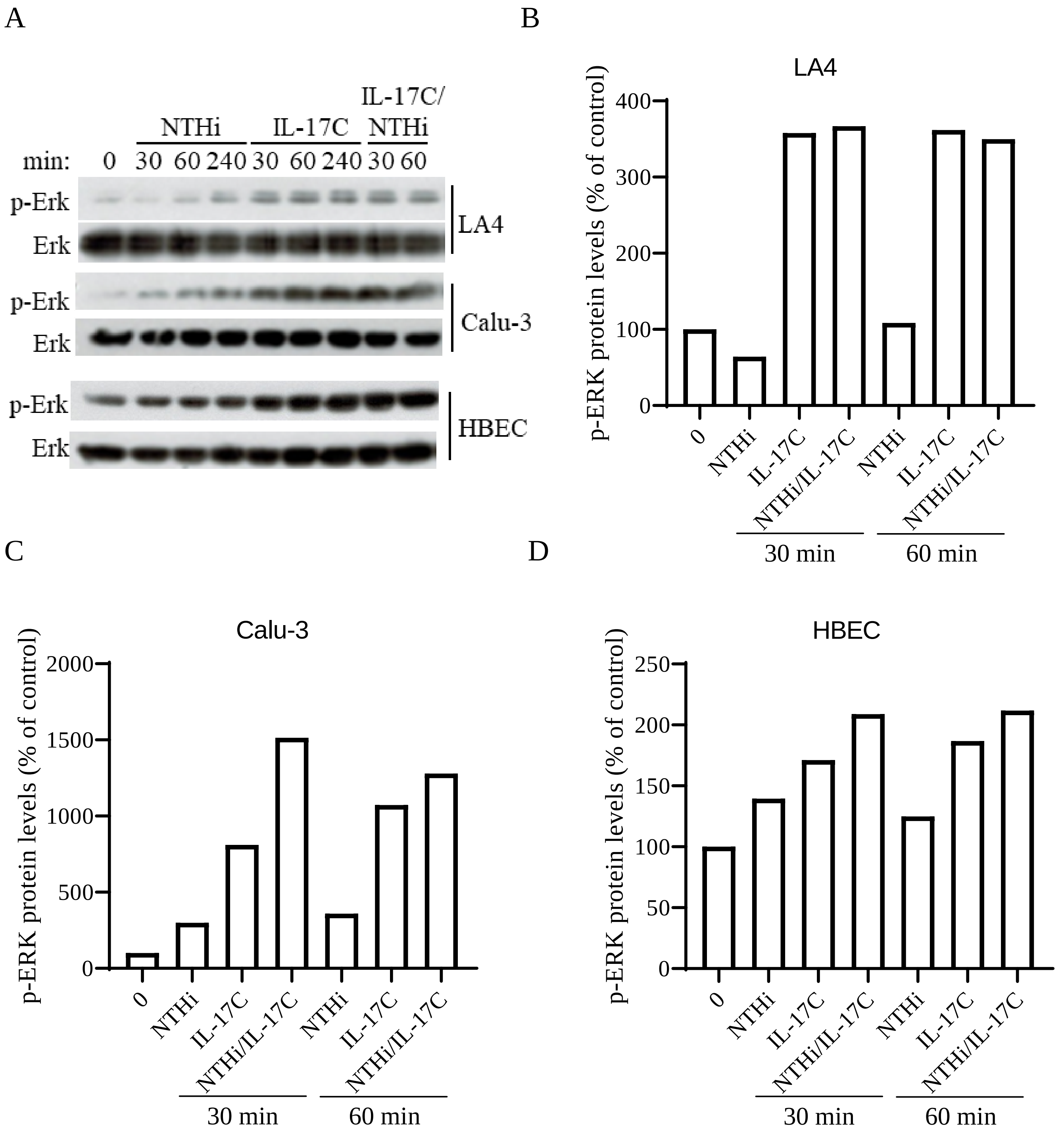

S2: The cancer cell lines LA4 and Calu-3 and HBEC were stimulated with 100 ng/ml IL-17C or heat-inactivated NTHi (10^7 CFU/ml) for the indicated time points. (A) Cell lysates were gel separated and immunoblotted. (B to D) Semi-quantitative densitometry of P-ERK normalized to ERK.

Lanes:
1: NTHi, 0 min
2: NTHi, 30 min
3: NTHi, 60 min
4: NTHi, 240 min
5: IL-17C, 30 min
6: IL-17C, 60 min
7: IL-17C, 240 min
8: NTHi + IL-17C, 30 min
9: NTHi + IL-17C, 60 min

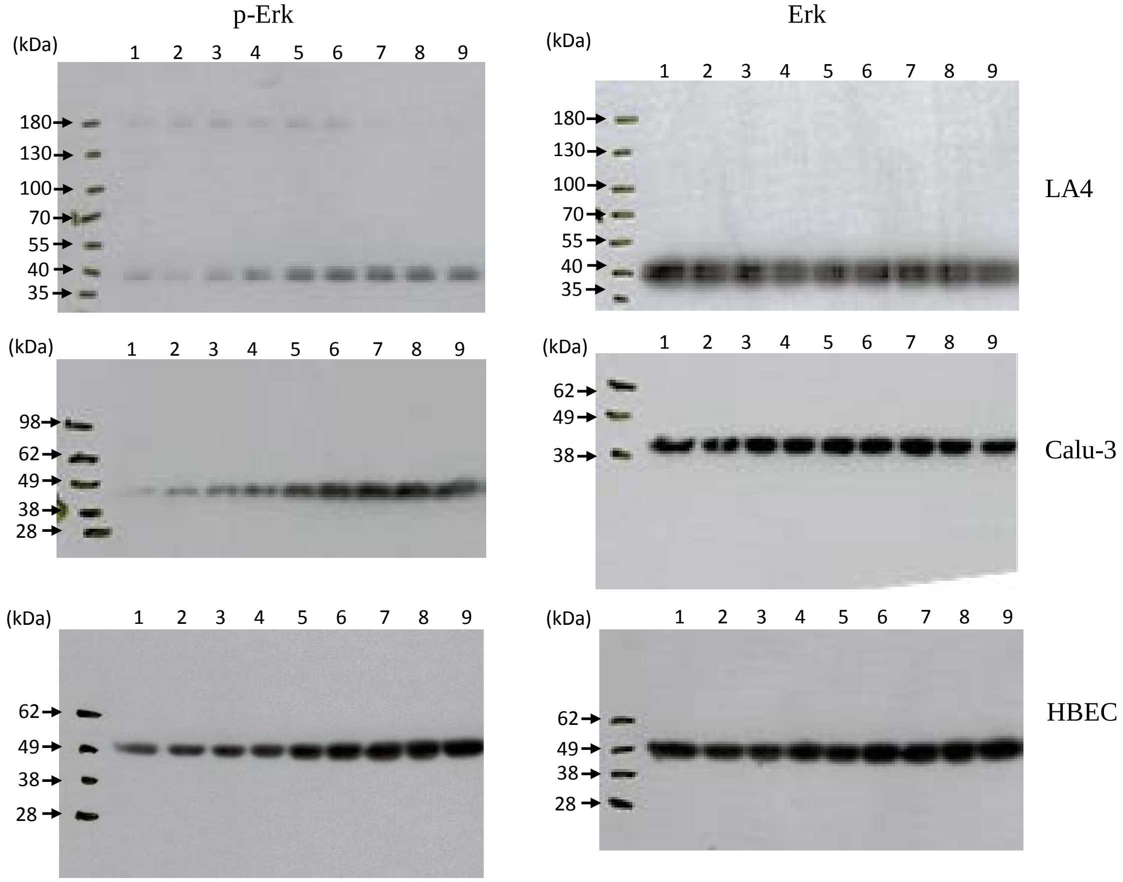

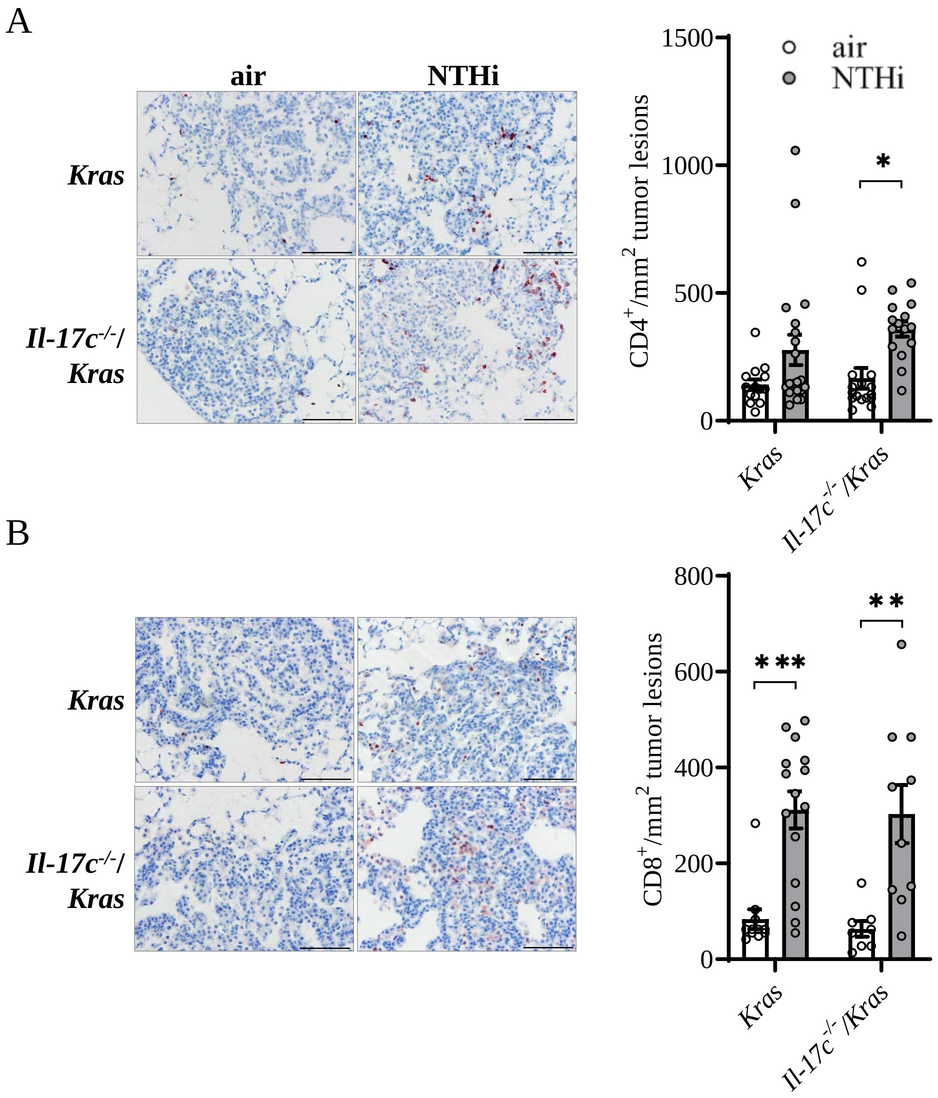

S3: *Kras* mice and *Il-17c*$^{-/-}$/*Kras* mice were exposed to NTHi for 4 weeks. (A) IHC of CD4 and quantification of the CD4 positive cells in cancerous lesions. (B) Representative IHC of CD8 and quantification of the CD8 positive cells in cancerous lesions. Data were compared by Two-way ANOVA with Bonferroni post test and *$p < 0.05$, **$p < 0.01$, and ***$p < 0.001$.

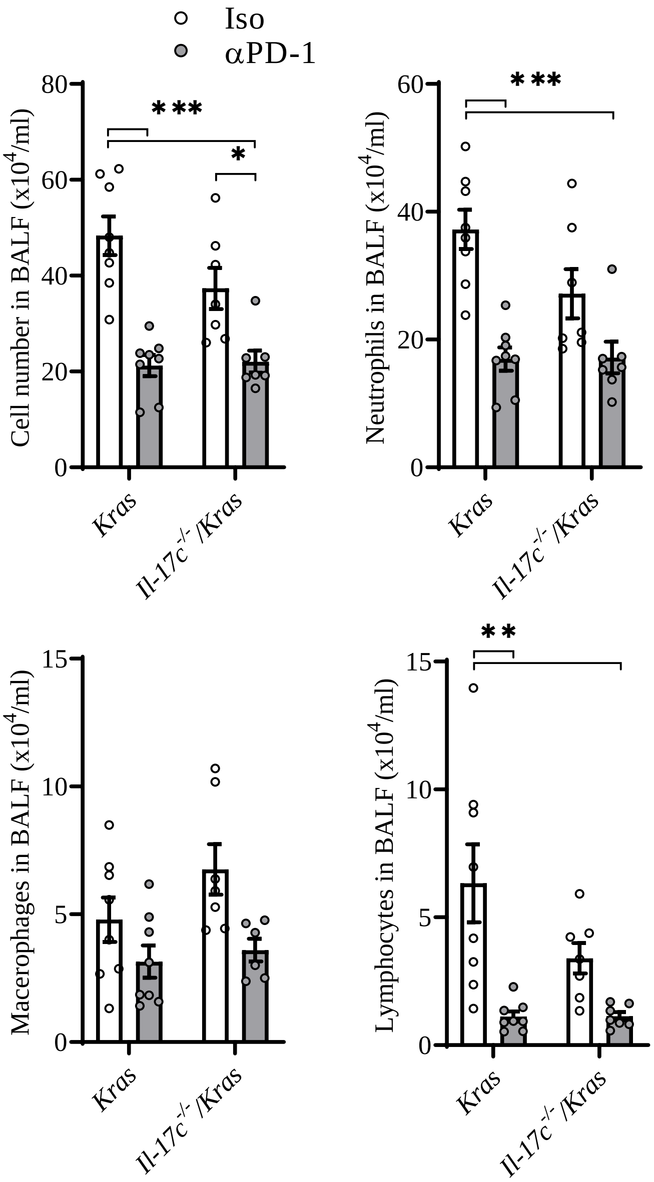

S4: *Kras* mice and *Il-17c*$^{-/-}$*/Kras* mice were exposed to NTHi for 4 weeks and treated with an anti-PD-1 antibody or an isotope antibody during the exposure phase. Numbers of total cells, neutrophils, macrophages, and lymphocytes were determined in BAL fluids Data were compared by Two-way ANOVA with Bonferroni post test and *p < 0.05, **p < 0.01, and ***p < 0.001.

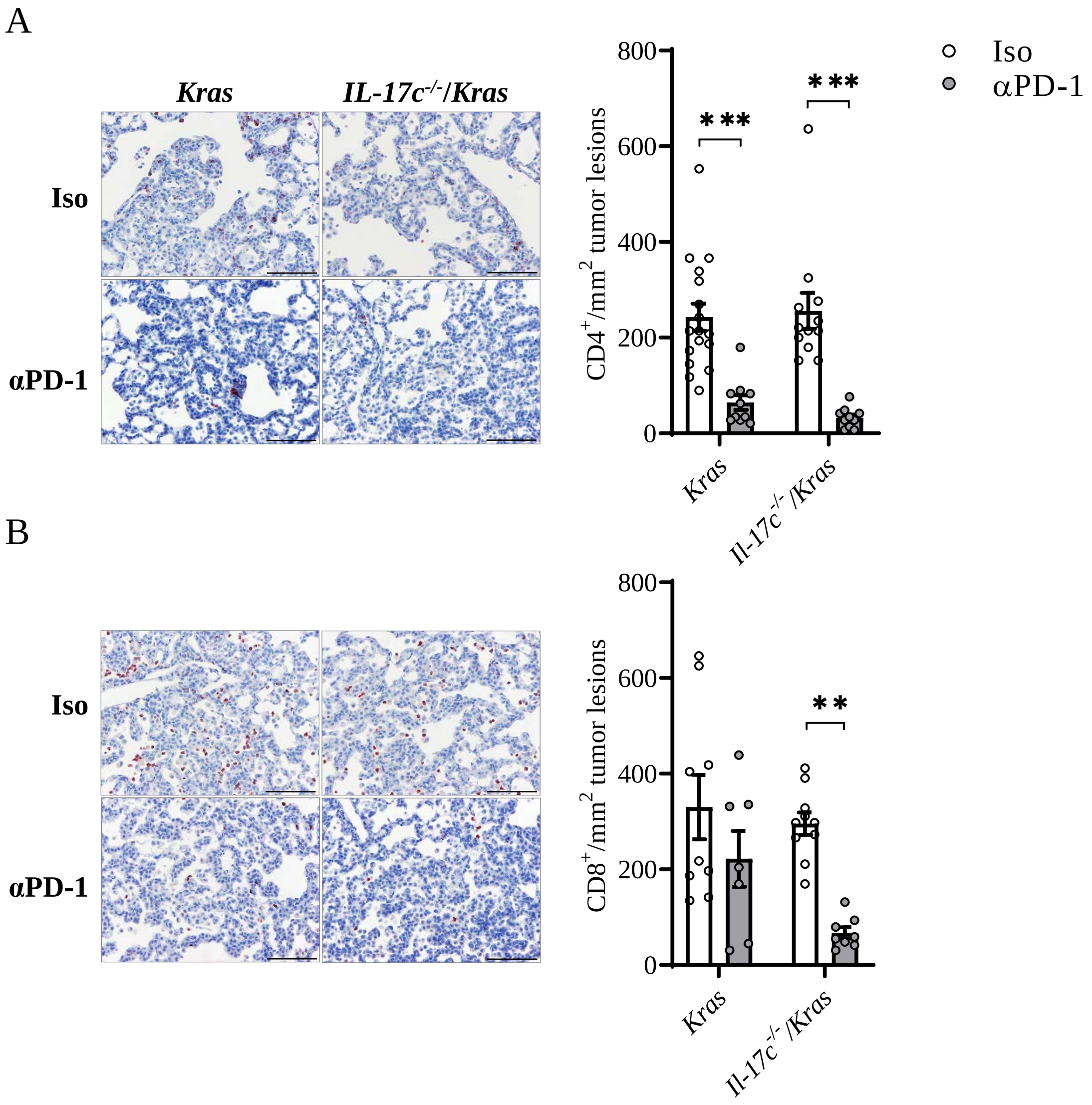

S5: *Kras* mice and *Il-17c*$^{-/-}$/*Kras* mice were exposed to NTHi for 4 weeks and treated with an anti-PD-1 antibody or an isotope antibody during the exposure phase. (A) Representative IHC of CD4 and quantification of the CD4 positive cells in tumor lesions. (B) Representative IHC of CD8 and quantification of the CD8 positive cells in tumor lesions. Data were compared by Two-way ANOVA with Bonferroni post test and are shown as the mean ± SEM. $^{**}p < 0.01$, and $^{***}p < 0.001$.
